# Familial Hypercholesterolaemia and the Risk of Cardiovascular Events

**DOI:** 10.7759/cureus.97656

**Published:** 2025-11-24

**Authors:** André Conchinha, Afonso Rodrigues, Tiago Pack, Sofia Cunha, António Santos

**Affiliations:** 1 Internal Medicine, Centro Hospitalar Universitário de Lisboa Central, Lisbon, PRT; 2 Internal Medicine, Medicina 4 - Hospital de Santa Marta, Unidade Local de Saúde de São José, Lisbon, PRT; 3 Internal Medicine, Unidade Local de Saúde de São José, Lisbon, PRT

**Keywords:** dyslipidaemia, familial hypercholesterolaemia, ldl receptor, low-density lipoprotein cholesterol (ldl), statins

## Abstract

Familial hypercholesterolaemia (FH) is an autosomal dominant genetic disorder, characterised by markedly elevated levels of low-density lipoprotein cholesterol (LDL-C) from birth, which confers a substantially increased risk of premature atherosclerotic cardiovascular disease (ASCVD).

Pathogenic variants primarily occur in the genes encoding the low-density lipoprotein receptor (LDLR), apolipoprotein B (ApoB), low-density lipoprotein receptor adaptor protein 1 (LDLRAP1), or proprotein convertase subtilisin/kexin type 9 (PCSK9).

Early diagnosis, based on clinical criteria, family history, and genetic testing, is imperative to promptly initiate aggressive therapeutic strategies.

Standard treatment involves lifestyle modifications and high-intensity pharmacotherapy, primarily with statins, often in combination with ezetimibe. For patients who do not achieve their therapeutic goals or are intolerant, PCSK9 inhibitors represent a significant evolution in the treatment paradigm.

In this article, we present a case of homozygous familial hypercholesterolaemia.

## Introduction

Familial hypercholesterolaemia (FH) is one of the most common monogenic diseases, with an estimated prevalence of one in 250-300 individuals for the heterozygous form (HeFH) and one in 160,000-300,000 for the much rarer and more severe homozygous form (HoFH). The condition is defined by the dysfunction of LDL-C metabolism, resulting in its chronic accumulation in the plasma and deposition in tissues, namely, in the arterial walls, leading to accelerated atherosclerosis, and in extravascular tissues, forming xanthomas and early development of corneal arcus [1-4].

Cholesterol homeostasis is rigorously regulated, with the low-density lipoprotein receptor (LDLR) pathway being the primary mechanism for clearing circulating low-density lipoprotein cholesterol (LDL-C). In FH, genetic variants compromise this pathway, leading to patients having lifelong elevation of atherogenic cholesterol [1,2]. Without treatment, it is estimated that 50% of men and 30% of women with HeFH will develop coronary artery disease (CAD) before the ages of 50 and 60, respectively. Early identification and treatment are, therefore, crucial to alter the natural history of the disease and reduce cardiovascular morbidity and mortality [1,3,4].

## Case presentation

We report a case of a 19-year-old female with no significant past medical history. As for family history, the mother had dyslipidaemia and died at age 47 from acute coronary syndrome (ACS), and the maternal grandfather had a history of hypertension and dyslipidaemia and died at age 50 from ACS. On the paternal side, first and second-degree relatives had dyslipidaemia, and the grandmother had an ACS at the age of 60. On physical examination, the patient presented with arcus cornealis and xanthelasma.

**Figure 1:**
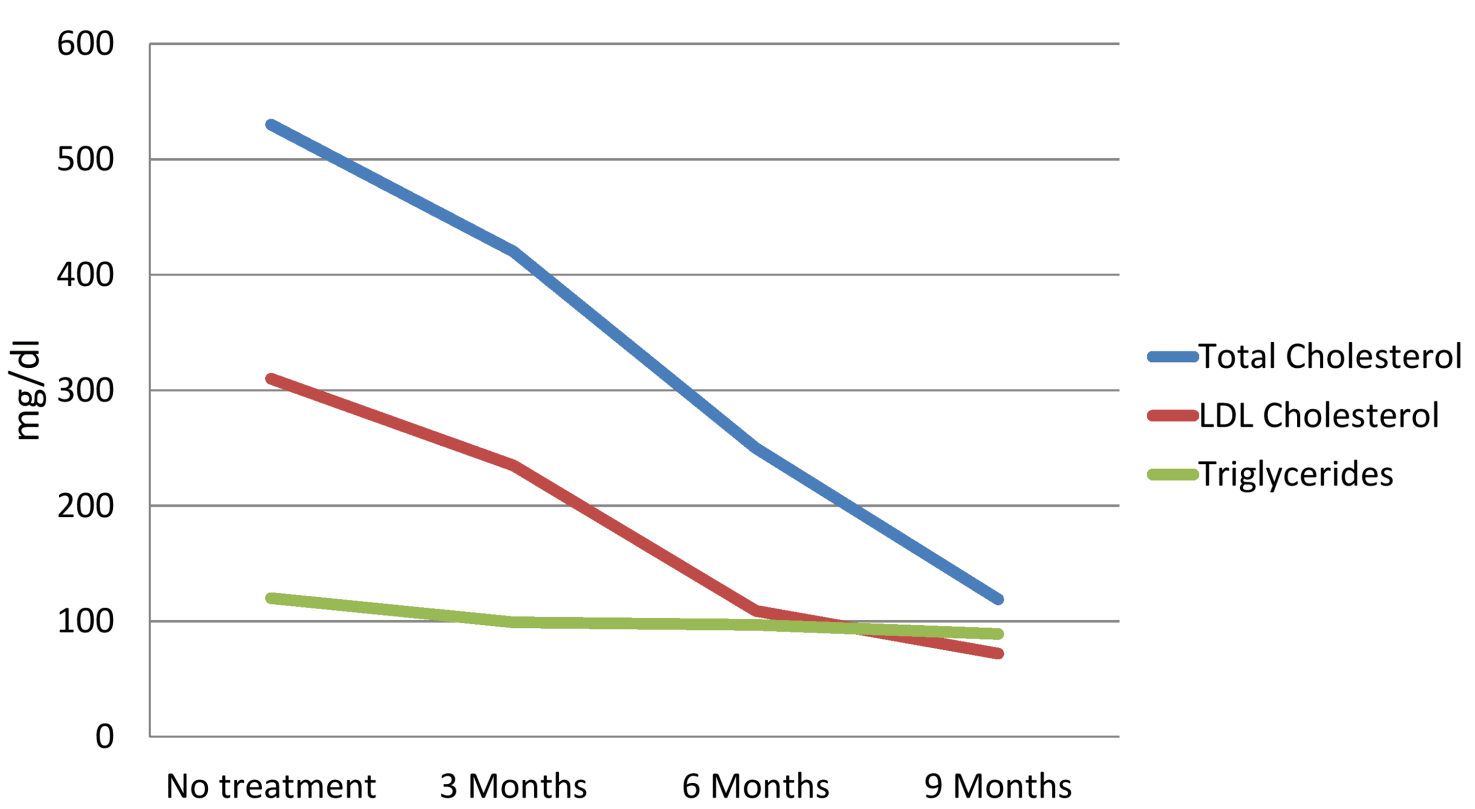
Evolution of the lipid profile according to treatment over time LDL: low-density lipoprotein.

Considering the history and the aforementioned findings, the general practitioner requested a lipid profile with the following results: total cholesterol (TC) = 530 mg/dL, LDL-C = 310 mg/dL, and triglycerides (TG) = 120 mg/dL. In this context, the general practitioner referred the patient to an internal medicine clinic, and therapy with rosuvastatin 20 mg and ezetimibe 10 mg was initiated (Table 1 and Figure 1). The remaining analytical evaluation, including thyroid and liver function tests, showed no abnormalities.

**Table 1:** Evolution of the lipid profile with therapy over time. TC: total cholesterol; LDL: low-density lipoprotein; TG: triglycerides; iPCSK9: proprotein convertase subtilisin/kexin type 9 inhibitors.

	No treatment	Rosuvastatin 20 mg + ezetimibe 10 mg (3 months)	Rosuvastatin 20 mg + ezetimibe 10 mg + iPCSK9 (9 months)	Unit	Reference value
TC	530	420	119	mg/dl	<190
LDL	310	235	72	mg/dl	<115
TG	20	99	89	mg/dl	<150

In the internal medicine clinic, the previously instituted therapy was maintained, and a genetic study was arranged, which documented a homozygous mutation in the LDLR gene. After three months of treatment, a reduction in the lipid profile was observed: TC = 420 mg/dL, low-density lipoprotein (LDL) = 235 mg/d, and TG = 90 mg/dL. Considering this is a case of FH, it confers a high cardiovascular risk, for which the LDL target is 70 mg/dL. In this regard, additional therapy with a proprotein convertase subtilisin/kexin type 9 (PCSK9) inhibitor (evolocumab) was initiated. After six months of triple therapy, the lipid profile was as follows: TC = 119 mg/dL, LDL = 72 mg/dL, TG = 89 mg/dL (Figure 1 and Table 1). The patient continued with biannual follow-up in the clinic with a stabilised lipid profile.

## Discussion

Mutations in the LDLR gene are responsible for more than 90% of FH cases. More than 2000 mutations have been described that affect the synthesis, transport, binding, or internalisation of the LDL receptor, resulting in an ineffective removal of LDL-C from the plasma [1,3,4].

The diagnosis of FH is predominantly clinical. The classic signs include analytical and clinical alterations, and a family history of cardiovascular events. Analytically, LDL-C levels are usually >190 mg/dL in adults or >160 mg/dL in children, in the absence of secondary causes. From a physical examination perspective, one might mention the following: tendon xanthomas (pathognomonic of FH, most frequently located on the Achilles and hand extensor tendons); arcus cornealis (lipid deposition on the periphery of the cornea, suggestive of FH if present before the age of 45); xanthelasmas (cholesterol deposits on the eyelids, but are not specific to FH). Regarding family history, the presence of hypercholesterolaemia and/or premature atherosclerotic cardiovascular disease (ASCVD) in first-degree relatives is suggestive of FH [1,5].

Scoring systems, such as the one from the Dutch Lipid Clinic Network (DLCN), the Simon Broome, or the Make Early Diagnosis to Prevent Early Death (MEDPED), are valuable tools that integrate these findings to stratify the probability of diagnosis (definite, probable, or possible) [1,6-8].

Considering the hereditary nature of the disease, cascade screening (identification of relatives of an index case) is the most cost-effective strategy for identifying new cases. Genetic testing, although not indispensable for a clinical diagnosis, is of great value as it confirms the diagnosis, allows for precise genetic counselling, improves therapeutic adherence, and may have prognostic implications [1,6].

The primary objective of treatment is the drastic reduction of LDL-C to decrease the lifelong cardiovascular risk. Lifestyle changes, namely, a diet low in saturated fats, combined with regular physical exercise, weight control, and smoking cessation, are paramount, although insufficient as monotherapy [2-4].

From a pharmacological point of view, the therapeutic arsenal includes the following options: (1) high-potency statins: these are the cornerstone of treatment (e.g., atorvastatin and rosuvastatin), which make it possible to achieve a reduction of at least 50% in baseline LDL-C levels. (2) Ezetimibe: ezetimibe inhibits cholesterol absorption in the small intestine and, when combined with statins, promotes additional reductions of 15-20% in LDL-C [2,6,9]. (3) Bempedoic acid: it is an oral small molecule that inhibits cholesterol synthesis by inhibiting the action of ATP-citrate lyase, a cytosolic enzyme upstream of the 3-hydroxy-3-methylglutaryl-coenzyme A reductase pathway. The single available dose (180 mg/day) reduces LDL-C levels by approximately 23% as monotherapy, approximately 18% when administered in the context of statin therapy, and by 38% when administered in a fixed-dose combination with ezetimibe [2,5,7,9,10]. (4) PCSK9 inhibitors (PCSK9i): monoclonal antibodies (e.g., evolocumab and alirocumab) that bind to PCSK9, preventing LDLR degradation, allowing for robust and sustained reductions in LDL-C (an additional 50-60%) and have been shown to reduce cardiovascular events in large-scale studies [2-6]. (5) Inclisiran: a small interfering RNA (siRNA) therapeutic that lowers LDL-C by targeting hepatic production of PCSK9. Its mechanism of action involves conjugation to N-acetylgalactosamine (GalNAc), which facilitates selective uptake by hepatocytes via the asialoglycoprotein receptor. Once inside the hepatocyte cytoplasm, inclisiran is incorporated into the RNA-induced silencing complex (RISC). The guide strand of inclisiran directs RISC to bind specifically to PCSK9 mRNA, resulting in its catalytic cleavage and subsequent degradation [11]. (6) Monoclonal antibody inhibitors of angiopoietin-like protein 3 (ANGPTL3) lower atherogenic lipoproteins by antagonising ANGPTL3-mediated inhibition of lipase activity, thereby promoting lipoprotein clearance. This mechanism is independent of LDL receptor function, which is clinically significant for patients with homozygous FH, where LDL receptor activity is absent or severely impaired. (7) Evinacumab, a monoclonal antibody targeting ANGPTL3, exemplifies this approach and has demonstrated substantial reductions in LDL cholesterol and triglycerides in both animal models and human studies [12]. (8) Microsomal triglyceride transfer protein (MTP) inhibitors act by blocking the activity of MTP, a heterodimeric protein located in the lumen of the endoplasmic reticulum of hepatocytes and enterocytes. MTP is essential for the transfer of triglycerides, cholesteryl esters, and phospholipids onto apolipoprotein B (apoB), a critical step in the assembly and secretion of very-low-density lipoprotein (VLDL) in the liver and chylomicrons in the intestine. The reduction in LDL-C is primarily due to a decreased rate of production of apoB-containing lipoproteins, rather than increased clearance [13]. (9) Lipoprotein apheresis: an extracorporeal procedure to remove LDL-C from the plasma, reserved for patients with HoFH or very severe cases of HeFH who do not respond to pharmacotherapy [2,6,10].

The treatment of FH during pregnancy is primarily non-pharmacological, with pharmacological options reserved for select high-risk cases. The mainstay of management is a heart-healthy lifestyle, including a diet low in saturated and trans fats, increased soluble fibre, and avoidance of smoking. Statins are contraindicated in pregnancy due to concerns about teratogenicity and should be discontinued prior to conception. For women with severe FH (especially homozygous FH or those with established ASCVD), bile acid sequestrants (such as colesevelam or cholestyramine) are considered the safest pharmacological option, as they are not systemically absorbed and are generally regarded as safe in pregnancy, though they may reduce absorption of fat-soluble vitamins and require monitoring. In cases of very high cardiovascular risk or homozygous FH, LDL apheresis may be considered if available. Other lipid-lowering agents (PCSK9 inhibitors, ezetimibe, lomitapide, bempedoic acid) lack sufficient safety data for use in pregnancy and are not recommended. In this sense, discussing pregnancy planning is crucial in any female patient with childbearing potential and assuring appropriate birth control and a plan to reassess medications at the time of planned or unplanned pregnancy [14,15].

## Conclusions

FH is a high cardiovascular risk condition that remains underdiagnosed and undertreated globally. The therapeutic approach has evolved significantly, with the combination of statins, ezetimibe, and PCSK9 inhibitors, allowing the majority of patients with HeFH to be within the recommended lipid targets. The effective management of FH is a paradigm of preventive medicine, illustrating how early, genetically based intervention can mitigate the risk of potentially fatal cardiovascular events.
